# Evaluation of the Impact of Policymakers’ Decisions in the Management Capacity of Protected Areas: Efficiency Evidence from Five Countries

**DOI:** 10.1007/s00267-022-01729-1

**Published:** 2022-11-19

**Authors:** Juan Carlos Valdivieso, Paul F. J. Eagles, Joan Carles Gil

**Affiliations:** 1 Instituto de Patrimonio y Turismo Sostenible, Universidad San Francisco de Quito USFQ, Quito, Ecuador; 2 Department of Recreation and Leisure Studies, University of Waterloo, Waterloo, ON Canada; 3 Department of Management, Universitat Politècnica de Catalunya, Barcelona, Spain

**Keywords:** Efficiency evaluation, National parks, Management capacity, Data envelopment analysis, Tourism

## Abstract

Protected areas play an important role in biodiversity conservation and tourism. Significant efforts have been made to increase the amount of protected lands. A problem of increasing the amount of public protected areas is that governments and other institutions face difficulties in providing the necessary resources for effective management. Accordingly, managers must be as efficient as possible but the lack of comparative methods makes the evaluation of efficiency difficult. Using Data Envelopment Analysis, a non-stochastic and non-parametric approach, information from 29 protected areas in 5 countries was analyzed to compare management efficiency amongst them. The first result found is the level of management efficiency that each park has in comparison with the others parks. The other important result is a prediction of the changes in the outputs if there is a hypothetical budget change. These results allow the generation of information for decision making.

## Introduction

“Protected areas are the cornerstone of biodiversity conservation; they maintain key habitats, provide refugia, allow for species migration and movement, and ensure the maintenance of natural processes across the landscape. Not only do protected areas secure biodiversity conservation, they also secure the well-being of humanity itself.” (Convention on Biological Diversity 2014).

Protected Areas management is the combination of legal, political, administrative, investigative, planned, protectionist, coordinated, interpretive or educational actions, which translate into a better use and performance of a protected area, and the fulfillment of its objectives (Cifuentes et al. 2000). There are four categories of management in protected area: i) natural resource management; ii) management of cultural resources; iii) visitor management; and, iv) community liaison and development management (Hockings et al. 2006). These categories face several management challenges due to the three existing functional elements: the ownership of the resources, the sources of income for the management, and the managing entity (Eagles 2008, 2009). Due to the limited resources and management problems that these areas face, a much higher level of management capacity is required than at present (Cifuentes et al. 2000).

Some key questions of interest are whether the responsible authorities have the capacity to effectively manage their protected areas and whether this management is being carried out on the ground (Hockings et al. 2006). Evaluation is an important part of the management process. It is easier for protected area (PA) managers to make better decisions if they have a clear understanding of the problems and their causes (Cifuentes et al. 2000). Therefore, it is essential to analyze the current situation of different PAs and to compare them with others. The challenge is that PAs are part of a connected and complex world and developing new management solutions require thinking about this complexity (McCool 2022).

Each PA is unique and poses difficulties in the identification and homogenization (Moore and Polley 2007), causing a breach between research and implementable solutions (Kates et al. 2001). For these reasons, many of the solutions are based on the management of each protected area by itself and not on a comparison with others. Different models have been developed and used to examine management capacity in PAs. There are more than 50 different methodologies for assessing management effectiveness (Leverington et al. 2010), either with field-applied or theoretical methodologies (Cifuentes et al. 2000; Hockings and Hobson 2000; Kothari et al. 1989; Shaw and Wind 1997; Thorsell 1982). The most commonly used methodologies described in that study are: RAPPAM (Ervin 2003), Management Effectiveness Tracking Tool (Stolton et al. 2003), and ProArca/CAPAS (Corrales 2004). Other approaches are Visitor Experience and Resource Protection (VERP) developed by National Park Service (Manning 2001), Ecosystem Management (Brussard et al. 1998), Cultural Ecosystem Services (Plieninger et al. 2013), Limits of Acceptable Change (Stankey et al. 1985) and Miradi, a software created by Conservation Measures Partnership to better meet conservation goals and objectives of conservation projects (Thinley et al. 2020).

It is imperative to develop a method for comparing management between different PAs, which in turn can help identify areas for management improvement. This standard model must allow an estimate of the resources necessary to establish effective management (Hockings et al. 2006), and an understanding of how decisions can affect the outcomes (Eagles et al. 2002).

To date, there is no comprehensive methodology and no theoretical frameworks proposed to evaluate every PAs that exist (Whitelaw et al. 2014). A model, in its simplest form, should make it possible to compare the outputs and outcomes of virtually any PA, but must be general and relatively insensitive to the particular needs of each one (Hockings et al. 2006).

Using the approach of management efficiency in State Park Agencies in the USA developed by Valdivieso et al. (2015), this paper develops a model that allows a comparison of different PAs and determines the level of management efficiency. It also allows a prediction of how a change in inputs can impact the management outputs. The Data Envelopment Analysis (DEA) approach is a non-stochastic and nonparametric methodology that has many benefits, especifically for comparisons amongst protected areas. This study evaluates 29 protected areas in 5 countries.

## Theoretical Analysis

In 1993, the Convention on Biological Diversity defined the Strategic Plan for Biodiversity 2011–2020 with 20 targets for protection and conservation of the nature. To achieve Objective 11 of the Aichi Biodiversity Targets to protect 17% of the Earth´s land surface (Convention on Biological Diversity 2010) many countries have promoted the creation of new protected areas. The creation of new protected areas has increased substantially over the last 100 years (IUCN 2012). Presently there are 253,359 terrestrial and inland protected areas covering 21.29 million km2 (UNEP-WCMC and IUCN 2022). Throughout history, there have been many lands that have been protected for different reasons.

These areas play an important role in the conservation of significant and endangered ecosystems worldwide. They are the fundamental basis for the promotion of biodiversity, ecosystem services and human well-being (Bertzky et al. 2012), as well as being one of the instruments to curb biodiversity loss (Convention on Biological Diversity 2014).They also provide livelihoods for nearly 1.1 billion people, are the main source of drinking water for over a third of the world’s largest cities, and are an important factor in ensuring global food security (Mulongoy and Babu Gidda 2008).

Despite the importance of these spaces, many government agencies and non-governmental organizations find it difficult to finance them with public resources (Adams et al. 2008; Saayman & Saayman 2006; da Silva et al. 2021; Whitelaw et al. 2014). This significant increase in the number of public protected areas is good news for protecting our planet, but they often end up as “paper parks” or “half-empty” forests due to poor management (Dharmaratne et al. 2000; Redford and Feinsinger 2001).

More than half of the protected areas are experiencing an erosion of biodiversity, which is alarmingly widespread (Laurance et al. 2012). Management of protected areas is profoundly difficult due to multiple and sometimes ambiguous mandates (Naughton-Treves et al. 2005). Despite some improvements in the protection of important areas, global biodiversity is decreasing significantly (Butchart et al. 2010), which shows that not only it is important to increase the number of protected areas worldwide, but that it´s crucial to improve the efficiency and effectiveness of their management. The challenge is to learn how to manage tourism growth, which generates income and jobs, while minimizing the negative impact that this sector has on the environment, conserving cultural heritage and local ecosystems (Blanke & Chiesa 2008; Valdivieso 2019).

Simply creating a protected area may not increase the protection of species (Craigie et al. 2010; Laurance et al. 2012) or habitats (Joppa and Pfaff 2011), unless there are effective means to conserve nature in the long term (Geldmann 2013). Well-managed protected areas contribute to the maintenance, not only, of healthy ecosystems and endangered species, but also provide multiple benefits for humans (Bertzky et al. 2012; Burbano et al. 2022).

Administrators of these areas face a multiplicity of challenges, each of which deserves research and the formation of a strategic response (Eagles 2013). Due to the difficulty in its identification (Moore and Polley 2007) there is no standard management model for PAs (Zafra-Calvo et al. 2017). A standard model should allow to estimate the adequate resources necessary to establish efficient management (Hockings et al. 2006), and provide managers with information on how their management compares to the park’s objectives. Besides this, managers want to know who is affected by a decision and how (Eagles and McCool 2002). These prevailing needs constitute the research scope of this paper.

### Efficiency in Protected Areas

The majority of studies and approaches related to management capacity in protected areas analyze their effectiveness (Leverington et al. 2010). These studies are of great importance to improve the management of these territories but does not analyze their efficiency. Over the years, the search for tools to analyze efficiency in these complex systems has been a constant. For example, Hays (1959) takes a journey from 1890 to 1920 analyzing the ideas and values of conservation leaders in their attempt to elaborate the concept of efficiency in resource management. Or the work of Pinchot (1910) in his book “The Fight for Conservation” to understand the importance of efficiency in conservation. To start, it is necessary to understand what is efficiency in PAs. Efficiency is making the best use of resources or the capability of acting or producing effectively with a minimum amount or quantity of waste, expense, or unnecessary effort (Eagles et al. 2010).

The use of an economic model to study efficiency has been widely used in different industries sectors like agriculture, banking, supply chain, and transportation. Emrouznejad and Yang (2018) identified 10,300 journal articles used DEA to study efficiency analyzing inputs and outputs. The problem with PA management is that it´s difficult to define and quantify the inputs and the outputs, such as tourism and ecological services.

This study focuses on the relationship between inputs and outputs that determine the factors of management efficiency in PAs (Geldmann 2013). Using the Protected Area Management Effectiveness (PAME) approach developed for the International Union for the Conservation of Nature (IUCN) and the World Commission on Protected Areas of IUCN-WCPA, this research establishes the inputs and outputs needed to study management efficiency. The PAME analyzes the “assessment of how well the protected area is being managed - primarily the extent to which it is protecting values and achieving goals and objectives” (Hockings et al. 2006).

## Empirical Analysis

### Data Collection

The evaluation of management efficiency in PAs has many challenges and one is to obtain the information needed for the evaluation. Despite the importance of these areas, there is a lack of exhaustive, global information (Muñoz-Santos and Benayas 2012; Naughton-Treves et al. 2005). To collect the data needed to run the DEA model, we developed an extensive and specific survey. We obtained 29 completed responses from 5 different countries. The procedure is explained below.

Based on the inputs and outputs needed in this study (PAME), a data input form was developed using the survey generated by the National Association of State Park Directors (NASPD) as a reference. The form was placed on a webpage that allowed respondents to save the progress and resume input later. This webpage was developed in 4 languages (Spanish, English, French and Polish). Each form was completed by a park official through the website created for this purpose: www.parksmanagement.org.

All the data were from January to December 2013. The form consists of 8 tables, which allows a complete analysis of the parks. Initially, a pilot test was carried with a park to test all the procedures. For this test, the valuable collaboration of the Communal Natural Park of Les Valls del Comapedrosa (Andorra) was appreciated.

Due to the extensive information needed and the time constraints, we had difficulty convincing managers to give us the information. The Ministry of the Environment of Ecuador, the Ministry of the Environment of Poland and the Natural National Parks of Colombia acceded very graciously to contribute to the study and, thanks to their support; we were able to obtain information of many PAs on those countries. For the rest of the parks, it was the PA managers themselves who directly supported the process and completed the form. This task required a large number of emails and personal contacts with many people for the correct coordination of the data collection process.

The result of this data collection was 29 completed responses from 5 different countries: Andorra, Colombia, Ecuador, Poland, and Spain. Several others had to be discarded because they did not provide all the necessary information. The DEA requires data for all the variables studied.

### Construction of the Variables

The Protected Area Management Effectiveness is considered to have a greater "explanatory power", since it permits to examine the potential links between the performance of different components of the management cycle; such as the influence of budget or personnel on the outputs (Hockings et al. 2006). The outputs are the products and services provided by the action of management through a process while the outcomes are the achievements obtained. This approach focuses only on the relationship between inputs and outputs in protected areas (Table 1).

**Table 1:** List of inputs and outputs. Source: Hockings et al. (2006)

Inputs	Outputs
Staff numbers	Visitor numbers
Budget	Measures of the volume of workload
Infrastructure levels	Measures of physical outputs
Access to information	

For the variables in which more than one item has been used, the arithmetic mean has been calculated. For example, the total number of workers, the qualifications of the staff, and the number of benefits were used to get the staff variable. In order to calculate the arithmetic mean, the percentage of each of the fields was calculated, using as a reference the maximum value of all the parks analyzed. To choose the variables used in this analysis, we used the variables proposed in the PAME approach (Hockings et al. 2006).

### Inputs

#### Staff

It is necessary to take into account that, for this variable, not only the number of people who work was taken into account, but also their capabilities (Cifuentes et al. 2000; Valdivieso et al. 2021). First, a quantitative analysis was established, which is represented by the total number of existing staff: full-time staff, part-time staff, temporary staff and volunteers. In addition, a qualitative analysis was carried out, represented by two sectors: the qualification of the personnel (percentage of qualified personnel) and the economic benefits they have (number of benefits they have).

#### Budget

The budget data is the amount of money available to each protected area in 2013.

#### Infrastructure levels

The infrastructure data were divided into two parts:1) the number of square meters of construction within the PA and 2) the total number of facilities available to visitors. This variable was constructed using the framework of assessing management effectiveness (Hockings et al. 2006).

#### Access to information

Access to information was estimated by a staff member of the PA, comparing access to information for visitors and access to information for staff members. To do this, a scale of 1 to 10 was used.

### Outputs

#### Visitor numbers

This variable uses the amount of tourist visitors that arrive to each park (Hornback and Eagles 1999).

#### Workload

This output was divided into the number of control programs, the number of environmental programs, the number of patrols carried out, the total number of meetings with local communities and the number of legal actions instigated (Hockings et al. 2006).

#### Physical outputs

The physical output variable is the sum of the total kilometers of operational trails that exist in the park, the length of the boundaries of the park, number of brochures, and the area reforested in 2013. The variables chosen were taken from the framework for evaluating management effectiveness (Hockings et al. 2006).

### Methodology

This paper uses the Valdivieso et al. (2015) model developed to compare the management efficiency in state parks agencies in the United States of America. Developing some adaptation to using a similar methodology and obtaining information from 29 protected areas in 5 countries, this research provides a method to measure efficiency in PAs. The study has two phases that are explained above.

### Phase 1

The Data Envelopment Analysis (DEA) is a non-stochastic and non-parametric approach that uses linear programing algorithms to calculate the efficiency in relation to the production frontier established by the activity studied (Coelli et al. 2005).

There has been a significant growth in the number of publications using Data Envelopment Analysis between 1978 and 2016. The number of journal articles that use this methodology reached 10,300 by the end of 2016 (Emrouznejad and Yang 2018).

Considering *n* the number of DMUs (Decision Making Units), in this case the parks studied, that uses a number of inputs *m* and a number of inputs *s*. If we use *X*_*j*_ as the input vector for DMU_j_ where *X*_*j*_ = *(x1*_*j*_ *;…; xm*_*j*_*)*^*T*^, and *Y*_*j*_ as the output vector where *Y*_*j*_ = *(y1*_*j*_*; …; ys*_*j*_*)*^*T*^.

This study is output oriented using a constant return to scale (CRS) and variable return to scale (VRS). The maximum possible efficiency, represented by *θ´, is given by:*

$$\begin{array}{l} {\theta^\prime = \begin{array}{*{20}{c}} {\max \theta } \\ {\lambda \theta } \end{array}} \hfill \\ {s.t.} \hfill \\ {X^\prime \ge \mathop {\sum}\limits_{j = 1}^n {\lambda _jX_j} } \hfill \\ {\theta Y^\prime \le \mathop {\sum}\limits_{j = 1}^n {\lambda _jX_j} } \hfill \\ {\mathop {\sum}\limits_{j = 1}^n {\lambda _j = 1} } \hfill \end{array}$$

**Fig. 1:**
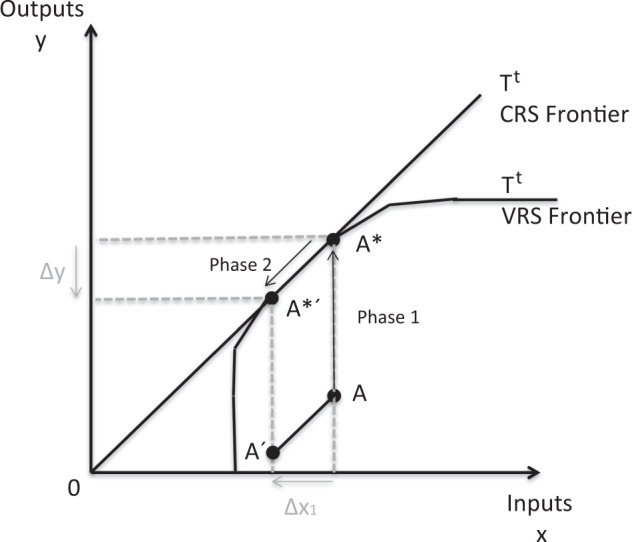
Graphic Explanation of the 2 phases of this study. Phase 1 is the graphic demonstration of the efficient point of this PA and Phase 2 is the change that will represent a policymaker decision. Source: self-made

The formula above explains the methodology used. The idea is to maximize the outputs (*Y*_*j*_) using the available inputs (*X*_*j*_). This requires taking into account all the variables of all the DMUs studied (*j*) and their possibility of production (*T*). DEA assigns weights (*λ*) to the inputs and outputs to obtain the best possible efficiency (*Θ´*), such that the sum of those weights is equal to 1. Therefore, if one DMU is at least as good as other DMUs, it gets a score of 1, otherwise it will be inefficient. Figure 1 shows the inefficiency of the DMU A and, compared to the other ones, it should increase the outputs until reaching the efficiency frontier (A*).

The distance between the outputs of a DMU and the technological frontier is called the distance function (Shephard 2012; Shephard et al. 1970). The results of the DEA analysis shows the distance between the current outputs of a unit and the optimal outputs that it could obtain compared to the others. Graphically, it will be the distance between point A and A* in Fig. 1.

This methodology has several advantages that are very important. For example, it is not necessary to obtain information about prices and costs. Another important advantage is that it is not necessary to make assumptions about the functional form of the variables and the data can be expressed in different units of measure.

It has been accepted, as a DEA convention, that the minimum number of DMUs analyzed should be greater than three times the sum of the amount of inputs used plus the number of outputs used in the model (Charnes et al. 1978; Raab and Lichty 2002). Another way to establish this number is by multiplying the number of inputs by the number of outputs used (Boussofiane et al. 1991). In this case, there are four inputs and three outputs which implies, under the approximations explained above, that the minimum number of protected areas that this study should evaluate is 21 for the first scenario or 12 for the second one. This study evaluates 29 parks, an adequate number to run the model.

### Phase 2

Once the study showed the efficiency level of each park in comparison to others, then the possible outcomes that each park might have in the case of a change in the inputs was calculated. The reasons for the changes could be, for example, a policymaker decision or a cut in the budget.

This study predicts the new outputs that a PA will have if there is a change of 5% of the budget or a change of 5% of the staff variable (number, qualification and economic benefit). These changes are based on the study of efficiency in State Parks Agencies of the USA (Valdivieso et al. 2015). This analysis can be replicated for other inputs. Figure 1 shows the idea of this section. If we take point A as the vector of inputs and outputs of a DMU A, A* will be its efficiency point at which the outputs should arrive. If there is a change in an input, in this case, a change in the budget (ΔX1), A’ would be the expected result that this DMU, in particular, will obtain.

The summary of the two phases is shown in Fig. 1. The first objective of a PA is to become efficient in comparison with the other units studied (A*). Once the efficiency has been reached, it is possible to predict the new outputs that would be obtained if there is a change in the inputs (A*´).

## Results and Discussions

This study produced an efficiency analysis for 29 protected areas from 5 different countries. For this comparative evaluation, 4 outputs and 3 inputs were used to study how managers are performing in comparison with the others parks. Table 2 shows a statistical summary of these variables. In this table, it is important to highlight some interesting data; one of them is the fact that the minimum number of users is zero. This is because some of the parks that have been analyzed have no visitors, because they have restricted access to the public. This is the case of the Las Hermosas National Natural Park in Colombia. Another fact to emphasize is that the data used are expressed in different units of measure. This is one of the greatest advantages when using DEA, since it does not require the use of the same unit of measure. The data delivered in euros were changed to US dollars, using a standard exchange rate, so that there is no variation in this aspect. The rest of the data can be seen in the Table 2.

**Table 2:** Descriptive statistics of the protected areas observed. Source: self-made

Category	Variable	Unit	Obs	Mean	Std. Dev.	Min	Max
**Inputs**	Staff	Percentage	29	40.24	16.51	12.06	76.28
	Budget	US Dollars (thousand)	29	2369.14	4486.57	61.29	20200.00
	Infrastructure	Percentage	29	11.01	18.68	0.02	99.52
	Information	Scale	29	6.22	1.89	1.00	8.50
**Outputs**	Users	Number (thousand)	29	118.42	283.64	0.00	1200.00
	Volume of work	Percentage	29	15.08	13.22	1.51	45.76
	Physical outputs	Percentage	29	15.02	13.69	0.63	51.19

To ensure the strength of the methodology used, we analyzed the correlation coefficient amongst the variables. For this study, we used the Pearson Correlation coefficient. This analysis ensures that the variables chosen, to determine the inputs and the outputs, have no correlation between each other. Correlation close to 1 determines a positive correlation and close to −1 is a negative correlation.

Table 3 shows the correlation matrix between the variables. The variables used in this study have no evidence of strong correlation between them and, therefore, it can be assumed that each input contributes to different aspects of management. It is interesting to observe that the budget does not show evidence of correlation with any of the other variable.

**Table 3:** Correlation matrix between the variables. *N* = 29 PAs. Source: self-made

	Staff	Budget	Infrastructure	Information	Users	Volume of work	Physical outputs
Staff	1.0000						
Budget	0.5202	1.0000					
Infrastructure	0.4116	0.7090	1.0000				
Information	0.2430	0.3030	0.1237	1.0000			
Users	0.3060	0.2880	−0.0196	0.3423	1.0000		
Volume of work	0.0124	0.3643	0.3656	−0.1598	0.3800	1.0000	
Physical outputs	0.3644	0.2962	0.1828	0.0202	0.1373	0.3441	1.0000

### Phase 1

Table 4 shows the results of this efficiency study. An efficient DMU (protected area for this study) is determined when the unit is located at the technological frontier or, what its’ equivalent, the efficient ones obtain a result equal to 1. This can be rationalized as they are obtaining, in comparison with the others PAs studied, the maximum possible outputs with their available inputs. As it can be seen on Table 4, there are some protected areas that are efficient and others that have large efficiency problems. An explanation for this result is that the methodology is constructing a technological frontier using the selected PAs at a specific time, which means that this is a specific analysis of this PAs in 2013. The model is not determining the optimal scenario.

**Table 4:** Distance function of the technical efficiency and the size of the protected areas studied. Source: self-made

		CRS Fo(x1,x2,x3,y1,y2,y3)	VRS Fo(x1,x2,x3,y1,y2,y3)	Size
Parc Natural Comunal de les Valls del Comapedrosa	Andorra	3.80	1.00	Small
Santuario de Fauna y Flora Iguaque	Colombia	5.74	5.45	Big
Parque Nacional Natural Tayrona	Colombia	1.00	1.00	Efficient
Parque Nacional Natural Las Hermosas	Colombia	1.19	1.00	Small
Parque Nacional Cajas	Ecuador	4.67	3.20	Big
Reserva de Producción de Fauna Cuyabeno	Ecuador	4.70	4.64	Big
Galapagos National Park 2013	Ecuador	1.87	1.17	Big
Parque Nacional Cotopaxi	Ecuador	1.00	1.00	Efficient
Parque Nacional Machalilla	Ecuador	1.00	1.00	Efficient
Parque Nacional Podocarpus	Ecuador	1.00	1.00	Efficient
Reserva Producción de Fauna Chimborazo	Ecuador	1.00	1.00	Efficient
Parque Nacional Yacuri	Ecuador	1.00	1.00	Efficient
Parque Nacional Cayambe Coca	Ecuador	1.24	1.23	Normal
Parque Nacional Sangay	Ecuador	1.01	1.00	Small
Reserva Ecológica El Angel	Ecuador	1.18	1.00	Small
Parque Nacional Llanganates	Ecuador	1.20	1.00	Small
Parque Nacional Sumaco Napo Galeras	Ecuador	1.38	1.00	Small
Reserva Ecológica Antisana	Ecuador	2.79	1.84	Small
Reseva Ecológica Cotacachi Cayapas zona baja	Ecuador	3.51	1.00	Small
Parc Nacional d’Aigüestortes i Estany de Sant Maurici	Spain	2.17	1.91	Big
Biebrzanski Park Narodowy	Poland	1.17	1.00	Big
Poleski Park Narodowy	Poland	3.15	2.44	Big
Drawieński Park Narodowy	Poland	3.71	2.76	Big
Woliński Park Narodowy	Poland	5.61	5.07	Big
Roztoczański Park Narodowy	Poland	5.75	4.52	Big
Kampinos National Park	Poland	1.00	1.00	Efficient
Wielkopolski Park Narodowy	Poland	1.00	1.00	Efficient
Gorczański Park Narodowy	Poland	1.00	1.00	Efficient
Babiogórski Park Narodowy	Poland	3.61	3.31	Small

*Fo* Farrell output-oriented measure of technical efficiency, *VRS* Variable return to scale, *Shaded rows* Efficient protected areas, *CRS* Constant return to scale

The results show that 5 parks in Ecuador, 3 in Poland and 1 in Colombia have optimum results; they are efficient (highlighted on Table 4), being located on the technological frontier. The closer the distance function is to 1, the closer they are to being efficient. This measure determines the level of optimal outputs that should be reached. For example, Poleski Park Naradowy in Poland has obtained a result of 3.15, so it would have to increase its outputs by 315% to reach maximum efficiency; this number is determined by the comparison with the other parks. This shows that this park is not obtaining the adequate outputs with the available inputs and, therefore, stakeholders of this park should focus on improving its outputs. On the other hand, the study shows that Parque Nacional Cotopaxi is efficient. These results should be taken with caution as each park has its own characteristics. Nevertheless, it is important that PAs stakeholders start to analyze the performance on their park in comparison with other ones.

In addition to the efficiency results, Table 4 shows the size of the analyzed park. The size column does not strictly refer to the size in square kilometers but to the amount of inputs they have. For example, the Parc de les Valls of Comapedrosa in Andorra is too small, this means that to reach the technological frontier, they would have to increase their outputs a lot, which is very complicated. On the other hand, if they decided to increase the inputs, it would not be difficult to achieve efficiency. This is due to economies to scale. There is a variable scale economy in management of protected areas (James et al. 1999) that could affect their efficiency.

### Phase 2

Phase 2 of the empirical investigation determines the changes that may occur with a theoretical change in the inputs. This phase contributes additional information for stakeholders. Table 5 reveals the impacts of an increase and a decrease of 5% in the budget and the staff variables.

**Table 5:** Predictions of future outputs in the case of a change in the inputs. Source: self-made

		Change in Y with 5% increase in		Change in Y with 5% decrease in	
		Staff	Budget	Staff	Budget
Roztoczański Park Narodowy	Poland	0.0%	0.0%	0.0%	0.0%
Parque Nacional Llanganates	Ecuador	0.0%	0.0%	0.0%	0.0%
Biebrzanski Park Narodowy	Polonia	0.0%	0.0%	0.0%	0.0%
Parque Nacional Natural Las Hermosas	Colombia	0.0%	0.0%	0.0%	0.0%
Parc Nacional d’Aigüestortes i Estany de Sant Maurici	España	0.0%	0.0%	0.0%	0.0%
Babiogórski Park Narodowy	Poland	0.0%	0.6%	0.0%	−0.6%
Parque Nacional Cayambe Coca	Ecuador	0.0%	0.9%	0.0%	−0.9%
Reserva de Producción de Fauna Cuyabeno	Ecuador	0.0%	2.4%	0.0%	−2.4%
Santuario de Fauna y Flora Iguaque	Colombia	0.0%	5.0%	0.0%	−5.0%
Drawieński Park Narodowy	Poland	0.2%	0.0%	0.0%	0.0%
Woliński Park Narodowy	Poland	0.2%	0.0%	−0.2%	0.0%
Galapagos National Park 2013	Ecuador	0.3%	0.2%	0.0%	0.0%
Reserva Ecológica Antisana	Ecuador	0.6%	1.8%	−0.6%	−1.8%
Parque Nacional Cajas	Ecuador	1.3%	0.0%	−1.3%	0.0%
Reseva Ecológica Cotacachi Cayapas zona baja	Ecuador	2.5%	2.5%	−2.5%	−2.5%
Reserva Ecológica El Angel	Ecuador	3.0%	2.0%	−3.0%	−2.0%
Parque Nacional Sangay	Ecuador	3.7%	1.3%	−3.7%	−1.3%
Parque Nacional Natural Tayrona	Colombia	3.8%	3.0%	−0.2%	−1.1%
Reserva Producción de Fauna Chimborazo	Ecuador	4.7%	5.0%	0.0%	−0.3%
Parque Nacional Cotopaxi	Ecuador	4.8%	5.0%	0.0%	0.0%
Gorczański Park Narodowy	Poland	5.0%	4.6%	0.0%	0.0%
Parque Nacional Machalilla	Ecuador	5.0%	5.0%	0.0%	0.0%
Parc Natural Comunal de les Valls del Comapedrosa	Andorra	5.0%	0.0%	−5.0%	0.0%
Parque Nacional Podocarpus	Ecuador	5.0%	2.2%	−2.8%	0.0%
Poleski Park Narodowy	Poland	5.0%	0.0%	−5.0%	0.0%
Kampinos National Park	Poland	5.0%	2.2%	0.0%	0.0%
Parque Nacional Yacuri	Ecuador	5.0%	4.6%	0.0%	0.0%
Wielkopolski Park Narodowy	Poland	5.0%	4.4%	0.0%	0.0%
Parque Nacional Sumaco Napo Galeras	Ecuador	5.0%	0.0%	−5.0%	0.0%
	**Average**	**2.4%**	**1.8%**	**−1.0%**	**−0.6%**

The bold values are the average of PAs

Table 5 reveals that there is a different impact for each park. Each column shows the predicted output change that will occur if there is a change in the budget or the staff. For example, Parque Nacional Natural Tayrona could increase its output by 3.8% if there is an increase of 5% in the staff variable. On the other hand, if there is an increase of 5% in the budget, the predicted outputs will increase by 3%.

The results of this phase show that each park will be affected in a different proportion and policymakers should take into account these results to take make decisions. A 5% change in an input will affect each PA differently. This is the most interesting part of the research since each area behaves differently, due to the different characteristics of each PA. In general, it can be stated that the outputs would be more affected if there were a change in personnel rather than the budget. This allows us to suppose that the personnel of the parks have a greater influence on the outputs than the budget. However, one cannot have an increase in the number of park staff without an increase in the budget. The results of this investigation shows similar results to Valdivieso et al. (2015) that studied management efficiency in State Parks Agencies in the United States of America.

## Conclusions and Limitations

Protected areas are important in preserving endangered ecosystems and countries are increasing in number. Around 15.78% of the terrestrial land is currently under a system of protection (UNEP-WCMC and IUCN 2022), but the global biodiversity continues to decline at an alarming rate (Butchart et al. 2010). It´s not enough to have the right number of PAs in the right place, it is also necessary to ensure that their governance is able to manage them in an effective manner and produce the desired outcomes (Dearden et al., 2005).

The increase of management capacity is important for protected areas (Carey et al. 2000) and this research provides a model to evaluate manager´s performance of different protected areas through evaluating their efficiency. Comparing PAs has been a problem due to the uniqueness of each one. Using Data Envelopment Analysis, a proven model in other industries, this research adapts the mathematical approach to review the impact of inputs and outputs on management efficiency.

This study focuses on observing the relationship between inputs and outputs so that parks will obtain as many outputs as possible. But, in addition to that, it is necessary that these outputs are transformed into the results for which these areas were created. Therefore, it is not only necessary to have more outputs but also that they could help reaching the objectives. Although the relationship between the two is not being studied, it is evident that obtaining more outputs is more likely to obtain better results.

This study provides a tool to evaluate efficient management capacity in protected areas and to predict the impact of an input change. Parks managers have a constant clamor of insufficient budget (Leverington et al. 2010) but it has been difficult to prove the impact that the budget has on the outputs. This model provides a tool to understand the impact of policymakers´ decision in protected areas outputs. Using DEA model, it is possible to analyze the current status of the relationship between inputs and outputs. This generates an idea of the current situation of each administration in order to make better decisions in these complex systems.

The results obtained show that there are some parks that are less efficient and need to make some changes to attain the efficiency frontier. The evaluation of 29 different parks shows that 9 have achieved optimal efficiency results, which means that they have been able to get as much outputs as possible with the available resources However there are parks that have had very poor results. The directors of those parks will need to make immediate changes and increase their outputs. To determine whether parks’ have long term efficiency, it would be desirable to include time series data from many years. Another interesting result obtained in this study is the demonstrations that the variable staff has a bigger influence on the outputs than the budget itself. Therefore, it’s essential to pay closer attention to the number and quality of employees of the protected areas.

This research explains the prevailing need of improving management efficiency, as there is an increasing number of PAs and, therefore, governments face problems to finance all the PAs. This need forces managers to be efficient in order to reach the planned objectives with the limited budget. It is important that each manager knows how to manage their resources in the most appropriate way to achieve the expected results.

To improve the accuracy of the results, this model should be used to analyze a much bigger database of protected areas. It is also important to have a standard and unified method of collecting the information so each park will be evaluated at the same level. At the same time, it is important to understand the complexity of this model. The analysis is carried out using the data submitted by each of the protected area managers and a comparison is made amongst them. This model shows that it is possible to analyze efficiency in PAs but the results will vary depending on the amount of information obtained.

In addition, it is important to understand that the PAMA explicitly differentiates between outputs and does not compare the overall picture of PA management. The use of this model helps understanding this interaction between the studied variables, but doesn’t provide the theoretical maximum, showing the current state of the studied APs. This will help in having more information to take decisions in these important areas but it has to be complemented with other approaches to analyze the entire system.

## References

[CR1] Adams C, Seroa da Motta R, Ortiz R, Reid J, Ebersbach C, de Almeida P (2008). The use of contingent valuation for evaluating protected areas in the developing world: Economic valuation of Morro do Diabo State Park, Atlantic Rainforest, São Paulo. Ecol Econ.

[CR2] Bertzky B, Corrigan C, Kemsey J, Kenney S, Ravilious C, Besançon C, Burgess N (2012). Protected Planet Report 2012: Tracking progress towards global targets for protected areas.

[CR3] Blanke J, & Chiesa T (2008) The Travel & Tourism Competitiveness Index 2008: Measuring Key Elements Driving the Sector’s Development. In *The Travel & Tourism Competitiveness Report 2008: Balancing Economic Development and Environmental Sustainability* (pp. 3–26). World Economic Forum.

[CR4] Boussofiane A, Dyson R, Thanassoulis E (1991). Applied data envelopment analysis. Eur J Operational Res.

[CR5] Brussard PF, Reed JM, Tracy CR (1998). Ecosystem management: what is it really. Landsc Urban Plan.

[CR6] Burbano DV, Valdivieso JC, Izurieta JC, Meredith TC, Quiroga Ferri D (2022). “Rethink and reset” tourism in the Galapagos Islands: Stakeholders’ views on the sustainability of tourism development. Ann Tour Res Empir Insights.

[CR7] Butchart SHM, Walpole M, Collen B, Van Strien A, Scharlemann JPW, Almond REA, Baillie JEM, Bomhard B, Brown C, Bruno J (2010). Global biodiversity: indicators of recent declines. Science.

[CR8] Carey C, Dudley N, Stolton S (2000) Squandering Paradise?: The Importance and Vulnerability of the World’s Protected Areas. *WWF-World WIde Fund for Nature*.

[CR9] Charnes A, Cooper WW, Rhodes E (1978). Measuring the efficiency of decision making units. Eur J Operational Res.

[CR10] Cifuentes M. Izurieta A, de Faria HH (2000) Measuring protected area management effectiveness. *WWF, GTZ, IUCN*, *2*.

[CR11] Coelli TJ, Prasada Rao DS, O’Donnell CJ, Battese GE (2005) Data Envelopment Analysis. In *An Introduction to Efficiency and Productivity Analysis* (pp. 161–181). Springer.

[CR12] Convention on Biological Diversity (2010) Strategic Plan for Biodiversity 2011-2010 and the Aichi Biodiversity Targets. *Conference Of The Parties To The Convention On Biological Diversity*.

[CR13] Convention on Biological Diversity (2014) *Protected Areas – an overview*. Protected Areas – an Overview. http://www.cbd.int/protected/overview/

[CR14] Corrales L (2004). Midiendo el éxito de las acciones en las áreas protegidas de Centroamérica: Medición de la Efectividad de Manejo.

[CR15] Craigie I, Baillie J, Balmford A (2010). Large mammal population declines in Africa’s protected areas. Biol Conserv.

[CR16] Dearden P, Bennett M, Johnston J (2005). Trends in global protected area governance, 1992–2002. Environ Manag.

[CR17] Dharmaratne GS, Yee Sang F, Walling LJ (2000). Tourism potentials for financing protected areas. Ann Tour Res.

[CR18] Eagles PFJ (2008) Governance models for parks, recreation and tourism. In *Transforming parks and protected areas* (Vol. 1, pp. 39–61).

[CR19] Eagles PFJ (2009). Governance of recreation and tourism partnerships in parks and protected areas. J Sustain Tour.

[CR20] Eagles PFJ (2013). Research priorities in park tourism. J Sustain Tour.

[CR21] Eagles PFJ, Havitz M, McCutcheon B, Buteau-Duitschaever W, Glover T (2010). The perceived implications of an outsourcing model on governance within British Columbia provincial parks in Canada: A quantitative study. Environ Manag.

[CR22] Eagles PFJ, McCool S (2002) *Tourism in national parks and protected areas: Planning and management*. CABI. http://books.google.com/books?hl=en&lr=&id=95GEsabzYI0C&oi=fnd&pg=PR5&dq=Tourism+in+national+parks+and+protected+areas:+Planning+and+management&ots=Fi3zP1nAut&sig=KtFQ6Jq1irSjeTER5UbPey9PpS8

[CR23] Eagles PFJ, McCool S, Haynes CD (2002). Turismo sostenible en áreas protegidas.

[CR24] Emrouznejad A, Yang G (2018). A survey and analysis of the first 40 years of scholarly literature in DEA: 1978–2016. Socio-Economic Plan Sci.

[CR25] Ervin J (2003) *WWF: Rapid Assessment and Prioritizationof Protected Area Management (RAPPAM) Methodology*. http://www.citeulike.org/group/664/article/1065223

[CR26] Geldmann J (2013) Evaluating the effectiveness of protected areas for maintaining biodiversity, securing habitats, and reducing threats [University of Copenhagen]. In *PhD thesis*. http://scholar.google.es/scholar?q=jonas+geldmann&btnG=&hl=es&as_sdt=0,5#7

[CR27] Hays SP (1959) Conservation And The Gospel Of Efficiency: The Progressive Conservation Movement, 1890–1920. In *Conservation And The Gospel Of Efficiency*. University of Pittsburgh Press. 10.2307/j.ctt7zw8b4

[CR28] Hockings M, Hobson R (2000). Fraser Island World Heritage Area Monitoring and Management Effectiveness Project Report.

[CR29] Hockings M, Stolton S, Leverington F, Dudley N, Courrau J (2006). Evaluating Effectiveness: A Framework for Assessing Management Effectiveness of Protected Areas. Second edition.

[CR30] Hornback K, Eagles P (1999) *Guidelines for public use measurement and reporting at parks and protected areas* (IUCN, Ed.).

[CR31] IUCN, U.-W. (2012). The world database on protected areas (WDPA).

[CR32] James A, Green M, Paine J (1999) A global review of protected area budgets and staff. *World Conservation Monitoring Centre. WCMC Biodiversity Series*, *10*.

[CR33] Joppa LN, Pfaff A (2011). Global protected area impacts. Proc R Soc B: Biol Sci.

[CR34] Kates RW, Clark WC, Corell R, Hall JM, Jaeger CC, Lowe I, McCarthy JJ, Schellnhuber HJ, Bolin B, Dickson NM, Faucheux S, Gallopin GC, Grübler A, Huntley B, Jäger J, Jodha NS, Kasperson RE, Mabogunje A, Matson P, Svedin U (2001). Sustainability science. Science.

[CR35] Kothari A, Pande P, Singh S, Variava D (1989). Management of national parks and sanctuaries in India.

[CR36] Laurance W, Useche D, Rendeiro J (2012). Averting biodiversity collapse in tropical forest protected areas. Nature.

[CR37] Leverington F, Costa KL, Pavese H, Lisle A, Hockings M (2010). A global analysis of protected area management effectiveness. Environ Manag.

[CR38] Manning R (2001) Visitor experience and resource protection: A framework for managing the carrying capacity of National Parks. J Park Recreation Administr 19(1). https://search.ebscohost.com/login.aspx?direct=true&profile=ehost&scope=site&authtype=crawler&jrnl=07351968&AN=31722519&h=zJwEAkBJXE2jhO5k9yWEWDGHsaRQ2NJlKVZwsxpqaHFNbtoFtsHDlFSB90u%2BmiGgtpiuZvqAt1yigjAC3X2GRw%3D%3D&crl=c&casa_token=BrDOEkSYREMAAAAA:UzcSreGmg09OWbHywubiaqsvbygEilTWm5fHK3LVjyTScU3dDDVbHY-ylRZLzc88cTPeM28xqzF2fI0

[CR39] McCool SF (2022). Thinking like a system in the turbulent world of outdoor recreation management. J Outdoor Recreat Tour.

[CR40] Moore SA, Polley A (2007). Defining indicators and standards for tourism impacts in protected areas: Cape Range National Park, Australia. Environ Manag.

[CR41] Mulongoy KJ, Babu Gidda S (2008) *The Value of Nature: Ecological, Economic, Cultural and Social Benefits of Protected Areas*. http://agris.fao.org/agris-search/search.do?f=2009/XF/XF0809.xml;XF2008417366

[CR42] Muñoz-Santos M, Benayas J (2012). A proposed methodology to assess the quality of public use management in protected areas. Environ Manag.

[CR43] Naughton-Treves L, Holland MB, Brandon K (2005). The role of protected areas in conserving biodiversity and sustaining local livelihoods. Annu Rev Environ Resour.

[CR44] Pinchot G (1910) The Fight for Conservation. In *wps.pearsoncustom.com (Vol. 1)*. Doubleday. https://wps.pearsoncustom.com/wps/media/objects/2429/2487430/pdfs/pinchot.pdf

[CR45] Plieninger T, Dijks S, Oteros-Rozas E, Bieling C (2013). Assessing, mapping, and quantifying cultural ecosystem services at community level. Land Use Policy.

[CR46] Raab R, Lichty R (2002). Identifying subareas that comprise a greater metropolitan area: The criterion of county relative efficiency. J Regional Sci.

[CR47] Redford KH, Feinsinger P (2001) The half-empty forest: sustainable use and the ecology of interactions. In *Conservation of Exploited Species* (Vol. 6, pp. 370–400). Conservarion Biology Series-Cambridge.

[CR48] Saayman M, Saayman A (2006). Estimating the economic contribution of visitor spending in the Kruger National Park to the regional economy. J Sustain Tour.

[CR49] Shaw P, Wind P (1997). Monitoring the condition and biodiversity status of European Conservation Sites.

[CR50] Shephard RW (2012) Cost and production functions. In *Springer Science & Business Media (Vol. 194)*. Springer Science & Business Media.

[CR51] Shephard RW, Gale D, Kuhn HW (1970) *Theory of cost and production functions*. Princeton University Press.

[CR52] da Silva JMC, Dias TCA, de C, da Cunha AC, Cunha HFA (2021). Funding deficits of protected areas in Brazil. Land Use Policy.

[CR53] Stankey GH, Cole DN, Lucas RC, Petersen ME, Frissell SS (1985) The limits of acceptable change (LAC) system for wilderness planning. *He Limits of Acceptable Change (LAC) System for Wilderness Planning, INT-176*, 33. https://www.cabdirect.org/cabdirect/abstract/19861835269

[CR54] Stolton S, Hockings M, Dudley N, MacKinnon N, Whitten T, Leverington F (2003) *Reporting progress in protected areas: a site-level management effectiveness tracking tool*. World Bank/WWF Alliance for Forest Conservation and Sustainable Use. https://www.thegef.org/gef/sites/thegef.org/files/Docs/GEF_SP_1_Tracking_Toolrev.doc

[CR55] Thinley P, Norbu T, Rajaratnam R, Vernes K, Dhendup P, Tenzin J, Choki K, Wangchuk S, Wangchuk T, Wangdi S, Chhetri DB, Powrel RB, Dorji K, Rinchen K, Dorji N (2020). Conservation threats to the endangered golden langur (Trachypithecus geei, Khajuria 1956) in Bhutan. Primates.

[CR56] Thorsell JW (1982). Evaluating effective management in protected areas: An application to Arusha National Park, Tanzania.

[CR57] UNEP-WCMC, IUCN (2022) *Protected Planet: The World Database on Protected Areas (WDPA)*. www.protectedplanet.net

[CR58] Valdivieso JC (2019). Stakeholders’ motivation to adopt corporate social responsibility practices in the lodging industry in an island destination: Balearic Islands case study. Int J Tour Policy.

[CR59] Valdivieso JC, Eagles PFJ, Gil JC (2015). Efficient management capacity evaluation of tourism in protected areas. J Environ Plan Manag.

[CR60] Valdivieso JC, Tapia E, Endara P, Ramia D, Azanza C (2021). An exploratory study: The importance of human resources in hotel performance in the Galapagos Islands. J Hum Resour Hospitality Tour.

[CR61] Whitelaw PA, King BEM, Tolkach D (2014). Protected areas, conservation and tourism – financing the sustainable dream. J Sustain Tour.

[CR62] Zafra-Calvo N, Pascual U, Brockington D, Coolsaet B, Cortes-Vazquez JA, Gross-Camp N, Palomo I, Burgess ND (2017). Towards an indicator system to assess equitable management in protected areas. Biol Conserv.

